# Human T-cell leukemia virus type 2 post-transcriptional control protein p28 is required for viral infectivity and persistence in vivo

**DOI:** 10.1186/1742-4690-5-38

**Published:** 2008-05-12

**Authors:** Brenda Yamamoto, Min Li, Matthew Kesic, Ihab Younis, Michael D Lairmore, Patrick L Green

**Affiliations:** 1 Center for Retrovirus Research, The Ohio State University, Columbus, OH 43210, USA; 2 Department of Veterinary Biosciences, The Ohio State University, Columbus, OH 43210, USA; 3 Department of Molecular Virology, Immunology, and Medical Genetics, The Ohio State, University, Columbus, OH 43210, USA; 4 Comprehensive Cancer Center and Solove Research Institute, The Ohio State University, Columbus, OH 43210, USA

## Abstract

**Background:**

Human T-cell leukemia virus (HTLV) type 1 and type 2 are related but distinct pathogenic complex retroviruses. HTLV-1 is associated with adult T-cell leukemia and a variety of immune-mediated disorders including the chronic neurological disease termed HTLV-1-associated myelopathy/tropical spastic paraparesis. In contrast, HTLV-2 displays distinct biological differences and is much less pathogenic, with only a few reported cases of leukemia and neurological disease associated with infection. In addition to the structural and enzymatic proteins, HTLV encodes regulatory (Tax and Rex) and accessory proteins. Tax and Rex positively regulate virus production and are critical for efficient viral replication and pathogenesis. Using an over-expression system approach, we recently reported that the accessory gene product of the HTLV-1 and HTLV-2 open reading frame (ORF) II (p30 and p28, respectively) acts as a negative regulator of both Tax and Rex by binding to and retaining their mRNA in the nucleus, leading to reduced protein expression and virion production. Further characterization revealed that p28 was distinct from p30 in that it was devoid of major transcriptional modulating activity, suggesting potentially divergent functions that may be responsible for the distinct pathobiologies of HTLV-1 and HTLV-2.

**Results:**

In this study, we investigated the functional significance of p28 in HTLV-2 infection, proliferation, and immortaliztion of primary T-cells in culture, and viral survival in an infectious rabbit animal model. An HTLV-2 p28 knockout virus (HTLV-2Δp28) was generated and evaluated. Infectivity and immortalization capacity of HTLV-2Δp28 *in vitro *was indistinguishable from wild type HTLV-2. In contrast, we showed that viral replication was severely attenuated in rabbits inoculated with HTLV-2Δp28 and the mutant virus failed to establish persistent infection.

**Conclusion:**

We provide direct evidence that p28 is dispensable for viral replication and cellular immortalization of primary T-lymphocytes in cell culture. However, our data indicate that p28 function is critical for viral survival *in vivo*. Our results are consistent with the hypothesis that p28 repression of Tax and Rex-mediated viral gene expression may facilitate survival of these cells by down-modulating overall viral gene expression.

## Background

The human T-cell leukemia viruses (HTLV types 1–4) are classified as complex retroviruses and members of the genus *Deltaretrovirus *[1]. HTLV-1 and HTLV-2 infections are the most prevalent worldwide, whereas infections with HTLV-3 and HTLV-4 were discovered only recently in a very limited number of individuals in Africa [2,3]. Although people infected with HTLV have a persistent antiviral immune response, these patients fail to clear virally infected cells. A small percentage of HTLV-1-infected individuals develop adult T-cell leukemia (ATL), a CD4+ lymphocyte malignancy, and various lymphocyte-mediated inflammatory diseases such as HTLV-1 associated myelopathy/tropical spastic paraparesis (HAM/TSP) [4-7]. However, only a few cases of atypical hairy cell leukemia or neurologic disease have been associated with HTLV-2 infection [8-12]. HTLV-1 and HTLV-2 have the capacity to promote T-lymphocyte growth both in cell culture and in infected individuals; however, the mechanism by which the virus persists in the infected individual, ultimately resulting in the oncogenic transformation of T-lymphocytes, is not completely understood.

In addition to the *gag, pol, and env *genes that encode the structural and enzymatic proteins, HTLV encodes *tax/rex *and accessory genes from pX open reading frames (ORFs) located in the 3' region of the genome. Tax increases the rate of transcription from the viral long terminal repeat (LTR) [13-15] and modulates the transcription or activity of numerous cellular genes involved in cell growth and differentiation, cell cycle control, and DNA repair [16-20]. Compelling evidence indicates that the pleiotropic effects of Tax on cellular processes are required for the transforming or oncogenic capacity of HTLV [21-23]. Rex acts post-transcriptionally by preferentially binding, stabilizing and selectively exporting the unspliced and incompletely spliced viral mRNAs from the nucleus to the cytoplasm, thus controlling the expression of the structural and enzymatic proteins as well as virion production [24-26]. Although both Tax and Rex are key positive regulators essential for efficient viral replication and, ultimately, cellular transformation, it has been hypothesized that the unregulated expression of these genes would result in the death of the infected cell *in vivo *via the induction of apoptosis and/or host immune response.

Growing evidence indicates that the HTLV-1 p30 and the HTLV-2 p28 accessory proteins encoded by pX ORF II regulate HTLV gene expression and therefore may contribute to the pathobiology of the virus. The homology between p30 and p28 is limited with the N-terminal 49 amino acids of p28 sharing 77% identity with the C-terminal portion of p30 [27,28]. Using over-expression studies, we and others reported that the nuclear/nucleolar-localizing p30 or p28 (p30/p28) specifically bind to and retain *tax/rex *mRNA in the nucleus [29,30]. Furthermore, inhibition of *tax/rex *mRNA export by p30/p28 appears to be co-transcriptional and requires an interaction between p30/p28 and Tax complexes on the viral promoter, which facilitates the co-migration of p30/p28 with RNA pol II until the protein encounters the newly synthesized downstream RNA binding sequence [31]. In addition, Sinha-Datta et al. demonstrated that p30 and Rex form a ribonucleoprotein ternary complex specifically on the *tax/rex *mRNA, which is consistent with its selective nuclear retention [32]. Interestingly, p30 also has been shown to interact with transcriptional co-activators/acetyltransferases, p300/CBP and TIP60, displaying both positive and inhibitory transcriptional effects on viral and cellular promoters [33-37]. Unlike p30, p28 does not display any significant transcriptional regulatory activity [29-31] suggesting the possibility of distinct or additional functions. Together, these findings suggest that p30/p28 facilitates virus and/or infected cell survival by regulating viral gene expression.

Under standard cell culture conditions, p30 was dispensable for viral infection, replication and immortalization of T-lymphocytes *in vitro *[38]. *In vivo *studies using a rabbit model of infection have revealed that p30 is important for the establishment of persistent infection [39,40]. However, more recent identification of HTLV-1 Hbz, found on the opposite coding strand partially overlapping p30, makes precise interpretation of these studies difficult. HTLV-2 containing a large deletion of the 3' proximal pX region maintained the capacity to efficiently replicate in and transform primary T-lymphocytes in culture, but was significantly attenuated in inoculated rabbits [41,42]. However, the specific contribution of the HTLV-2 accessory gene products, particularly p28, to overall virus biology has not been determined.

In this study, we evaluated the functional role of p28 in the context of an HTLV-2 infectious molecular clone and determined its contribution to viral replication and viral-induced immortalization in cell culture as well as viral replication kinetics and persistence in inoculated rabbits. Our findings indicate that the loss of p28 and thus its documented repressive post-transcriptional regulatory effect on Tax/Rex was not sufficient to disrupt the capacity of the virus to immortalize primary T-lymphocytes in culture. However, in the *in vivo *rabbit infection model, a p28-defective HTLV-2 had reduced replication and ability to establish persistent infection. These results suggest that the posttranscriptional repression of retroviral gene expression by p28 down-modulates viral replication thereby directly affecting cell signaling and survival. In addition, p28 may facilitate immune escape by HTLV infected cells by preventing their recognition by the host immune response.

## Results

### Generation and characterization of the HTLV-2 p28 knockout mutant

**Figure 1:**
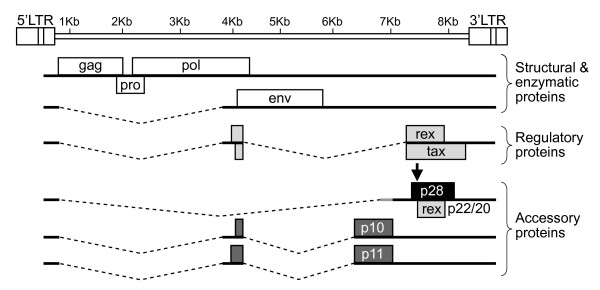
**Organization of the HTLV-2 genome and coding regions**. The complete proviral genome is shown schematically. Boxes denote long terminal repeats (LTRs). RNAs encoding the various protein ORFs are indicated. p28 has the potential to be encoded by two distinct singly-spliced mRNAs (gray line in p28 mRNA denotes utilization of two distinct splice acceptor sites). The arrow above p28 ORF depicts the location of the termination mutation to generate Δp28 (stop codon at amino acid 7).

**Figure 2:**
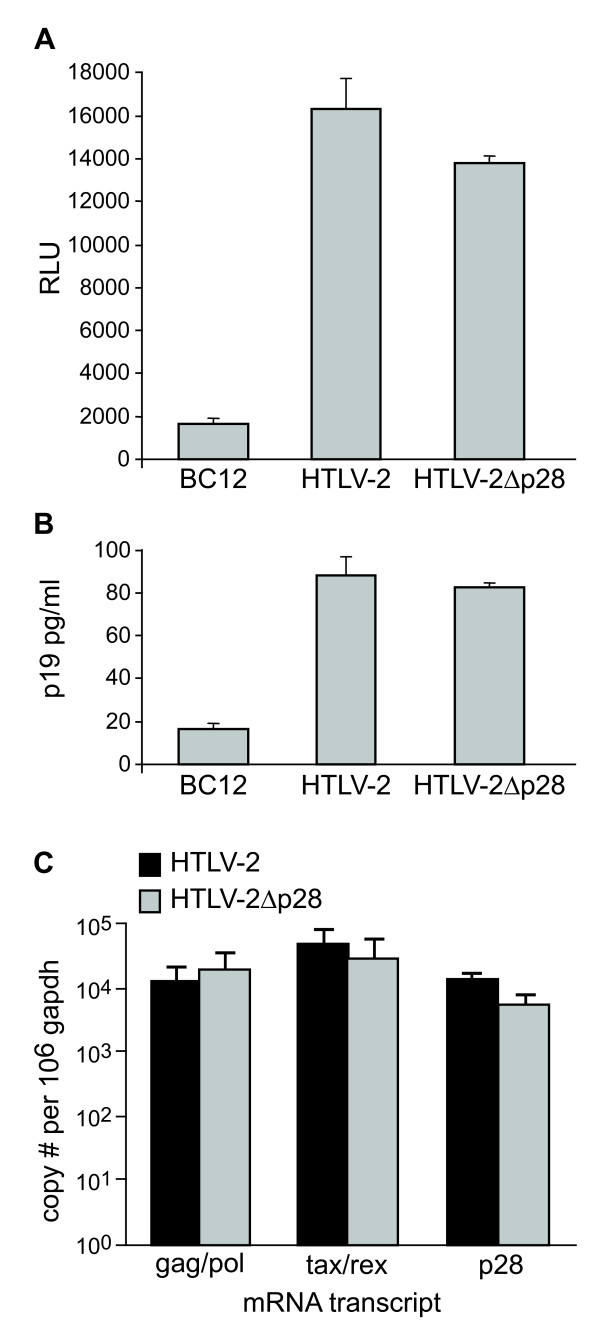
**Characterization of proviral clones *in vitro***. 293 T cells (2 × 10^5^) were co-transfected with 1 μg of wtHTLV-2 or HTLV-2Δp28 proviral clones or negative control DNA along with 0.1 μg of LTR-1-Luc and 0.01 μg of TK-*Renilla*. All transfections were performed in triplicate and normalized to TK-*Renilla *to control for transfection efficiency. Cell lysates or supernatants were harvested 48 h post-transfection. (A) Measure of Tax activity presented as relative luciferase units. Results indicated that loss of p28 expression from the proviral clone did not significantly alter Tax activity. (B) Rex activity as measured by expression of p19 Gag (virions) in the cellular supernatants. Results indicated that loss of p28 expression from the proviral clone did not significantly alter Rex activity. (C) Total RNA was extracted from 293 T cells transfected with HTLV-2 or HTLV-2pΔ28 as in panels A and B. mRNA copy number was quantified by Taqman realtime RT-PCR. The histogram represents the copy number of *gag-pol, tax/rex*, and p28 transcripts normalized to 1 × 10^6 ^copies of *gapdh*. Results indicated that deletion of p28 protein had no significant affect on *tax/rex*, *gag/pol*, or p28 mRNA expression.

**Figure 3:**
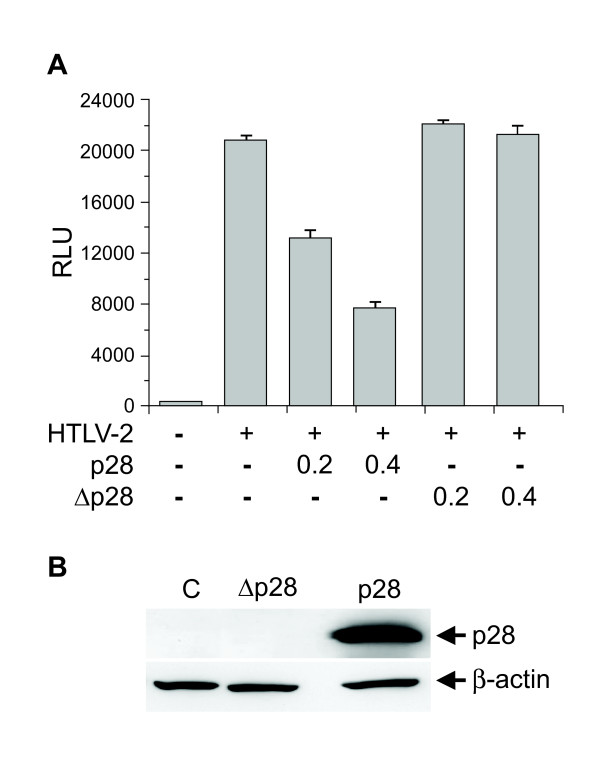
**Exogenously expressed p28, but not Δp28 results in dose-dependent repression of Tax-mediated transcription**. 293 T cells (2 × 10^5^) were co-transfected with 1 μg of wtHTLV-2 proviral clone or negative control DNA, 0.1 μg of LTR-2-Luc and 0.01 μg of TK-*Renilla*, and varying concentrations (0.2–0.4 μg) of CMVp28AU1 or CMVΔp28AU1 expression vectors as indicated. (A) Tax function was measured as firefly luciferase activity from LTR-2-Luc normalized to *Renilla *luciferase activity. RLU, relative light units. (B) Western blot analysis was performed on lysates to confirm expression of p28 (AU1 antibody) or β-actin as a loading control. As expected, results indicated that the Δp28 mutation disrupts p28 protein expression.

As a result of alternative splicing, HTLV-2 p28 has the potential to be expressed from two distinct singly-spliced mRNAs (Fig. 1). Both mRNAs also have the potential to produce the amino terminal truncated p22/p20 Rex proteins [28,43]. It is important to note that the p28 ORF has complete overlap with Tax exon 3 and partial overlap with Rex exon 3 (Fig. 1). Using an over-expression system approach, previous studies revealed that p28 is at least in part functionally homologous to HTLV-1 p30 and has the capacity to specifically retain *tax/rex *mRNA in the nucleus, thus decreasing Tax and Rex protein and viral replication via a posttranscriptional mechanism [30]. However, the specific role of p28 in the context of a proviral clone, and ultimately on virus biology, has not been investigated. In order to determine the potential role of p28 in HTLV-2-mediated cellular immortalization in cell culture and viral persistence in inoculated rabbits, a p28-deficient proviral clone (HTLV-2Δp28) was generated from the HTLV-2 molecular clone pH6neo. To construct HTLV-2Δp28, a single nucleotide was altered by site directed mutagenesis, which introduced a stop codon at amino acid 7 of the p28 ORF and had no affect on the overlapping Tax and Rex amino acid sequence. We initially determined whether knocking out p28 altered Tax and/or Rex activities. Co-transfection of wild-type HTLV-2 or HTLV-2Δp28, as a source of Tax, and the LTR-2-Luc reporter revealed that HTLV-2Δp28 had a consistently lower, but not significantly different LTR-directed gene expression (Fig. 2A). Moreover, cells transfected with HTLV-2Δp28 produced levels of p19 Gag in the culture supernatant similar to wild-type HTLV-2, indicating no significant repression of Rex function (Fig. 2B). Based on the reported functional activity of over-expressed p28, we were surprised that deletion of p28 did not translate into an increase in Tax activity or p19 Gag expression. Although p28 mRNA is easily detectable following transient transfection with proviral clones (Fig. 2C), we have been unable to detect p28 protein by Western blot [30] (Fig. 2C and data not shown). We then determined the effect of exogenously over-expressed p28- and Δp28-AU1 tagged proteins on Tax-mediated transcription. Our results confirmed previous reports that over-expressed p28 from a CMV-cDNA expression vector significantly repressed Tax activity in a dose-dependent manner (Fig. 3A). Importantly, the Δp28 cDNA expression vector failed to repress Tax activity (Fig. 3A). Western blot analysis confirmed the expression of p28 and that the Δp28 amino terminal truncation mutation resulted in a complete loss of p28 protein expression (Fig. 3B). Therefore, our results are consistent with the conclusion that either p28 protein is not expressed from the proviral clone following transient transfection (48 hours) or that the levels of p28 expressed from the proviral clone are below the threshold concentration required for detection by Western blot and necessary for repression of Tax or Rex activity.

### HTLV-2Δp28 promoted virus-induced proliferation and immortalization of PBMCs

**Figure 4:**
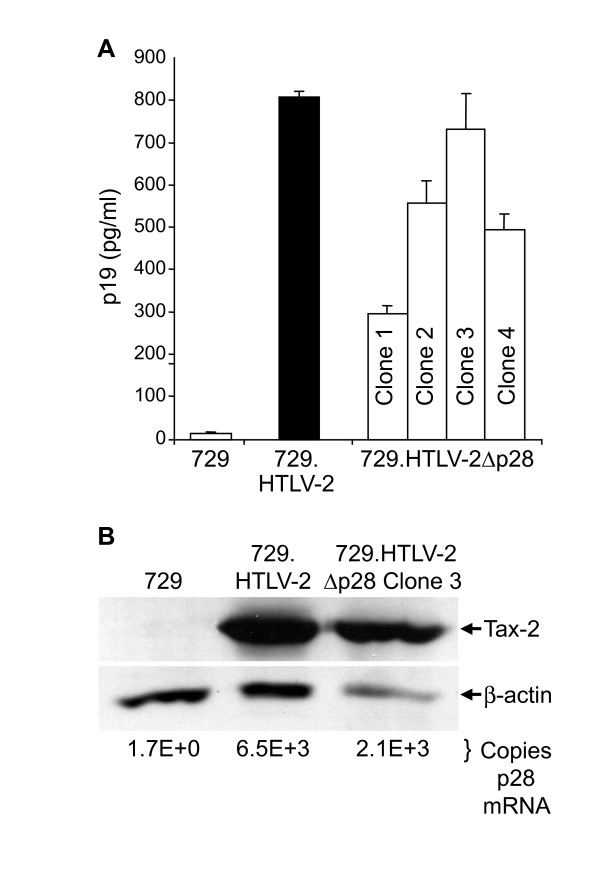
**Expression of p19 Gag and Tax protein and p28 mRNA in permanent transfectants**. (A) Four 729 stable transfectants (clone 1–4) were isolated for HTLV-2Δp28 as described in Materials and Methods. Our well-established 729pH6neo (729.HTLV-2) cell clone was used as the wtHTLV-2 stable producer cell line. p19 Gag was quantified by ELISA from the four independently isolated 729.HTLV-2Δp28 (Clones 1–4), 729.HTLV-2, and the 729 negative control. Each 729.HTLV-2 producer cell line displayed variable p19 production. (B) Clones indicated by asterisks, which have been shown to produce similar quantities of p19 Gag, were further characterized by Western blot for Tax protein expression using rabbit polyclonal antisera raised against Tax-2. β-actin was used as a loading control. Numbers below each lane are the copy number of p28 transcript per 10^6 ^copies of GAPDH determined by realtime RT PCR. The results show similar levels of p28 mRNA expression.

To determine the capacity of HTLV-2Δp28 to synthesize viral proteins, direct viral replication, and induce cellular immortalization, stable 729 cell transfectants expressing wild-type and p28-deleted HTLV-2 proviral clones were generated and characterized. Four independent stable HTLV-2Δp28 transfectants were isolated and found to contain complete copies of the provirus; the presence of the expected Δp28 mutation was confirmed by sequencing (data not shown). We quantified the concentration of p19 Gag produced in the culture supernatant of the four cell clones by ELISA. Our results showed p19 Gag expression ranging from 250–750 pg/ml (Fig. 4A). The variable p19 Gag expression from independent stable cell clones was attributed to chromosomal location of proviral sequences and overall proviral copy number, consistent with previous analyses [44,45]. We did not observe a pattern of increased viral gene expression in the absence of p28. For additional studies, we selected 729.HTLV-2Δp28Clone 3, a stable producer line with p19 Gag production similar to that of our well-characterized wild-type HTLV-2-producer cell line 729pH6neo (729.HTLV-2). Further characterization revealed that, as with transient transfection, p28 mRNA was detected at similar levels (approximately 10^3 ^copies per 10^6 ^copies of cellular gapdh mRNA) in 729.HTLV-2 and 729.HTLV-2Δp28Clone 3 (Fig. 4B), but Western blot analyses failed to detect p28 protein in 729.HTLV-2 or 729.HTLV-2Δp28 (data not shown). A similar level of Tax-2 expression in 729.HTLV-2 and 729.HTLV-2Δp28 relative to the β-actin loading control was detected by Western blot and, as expected, Tax-2 was not detected in the 729 negative control cells (Fig. 4B). Therefore, as with transient transfection, the repressive effect of p28 expressed from a stably integrated provirus on Tax-mediated transcription was not detectable.

**Figure 5:**
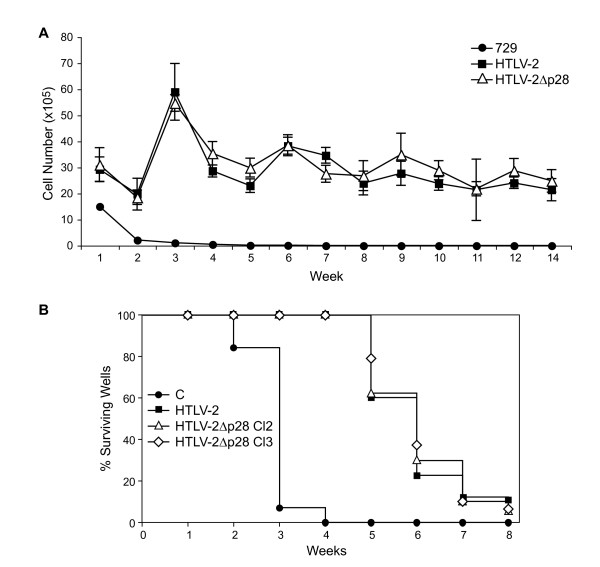
**p28 is dispensable for HTLV-2-mediated proliferation and immortalization of primary T-lymphocytes**. (A) Human PBMCs were isolated by Ficoll/Paque and co-cultivated with irradiated (10,000 rads) 729, 729.HTLV-2, or 729.HTLV-2Δp28 stable cell lines. PBMCs (2 × 10^6^) were cultured with irradiated donor cells (1 × 10^6^) in 24 well plates as indicated. A representative growth curve of HTLV-2 infected cells is shown. Cell viability was determined weekly by trypan blue exclusion (0–14 wks post co-cultivation). The mean and standard deviation of each time point was determined from three independent samples. (B) Pre-stimulated PBMCs (10^4^) were co-cultured with 2 × 10^3 ^irradiated 729 stable producer cells in 96 well plates. The percentages of proliferating wells were plotted as a function of time (wks). Representative Kaplan-Meir plots for wtHTLV-2, HTLV-2Δp28, and 729 uninfected control cells are shown. Results indicated that the percentage of wells containing proliferating lymphocytes was similar between wtHTLV-2 and HTLV-2Δp28 infected cells.

We assessed the ability of the HTLV-2Δp28 to induce proliferation and immortalize human PBMCs in co-culture assays. Freshly isolated human PBMCs co-cultured with lethally irradiated 729.HTLV-2 or 729.HTLV-2Δp28 in the presence of 10 U/ml of human IL-2 showed very similar progressive growth patterns consistent with the HTLV-2 immortalization process, whereas control cells died within the first few weeks (Fig. 5A). Immortalized PBMCs expressed similar levels of p19 Gag and harbored the expected HTLV-2 sequences indicating that viral transmission was responsible for the immortalization of PBMCs (data not shown). In an effort to obtain a more quantitative measure of the ability of these viruses to infect and immortalize PBMCs, a fixed number of PBMCs were co-cultured with virus-producing cells in a 96-well plate assay [45]. Since this assay is very stringent as a result of diluting the cultures 1:3 weekly, slowly growing or non-dividing cells are eliminated very quickly and the percentage of surviving wells is an accurate measure of the immortalization efficiency of viruses. A Kaplan-Meier plot of HTLV-2-induced T-cell proliferation or survival indicated that the percentage of wells containing proliferating lymphocytes was similar between HTLV-2 and two independently isolated HTLV-2Δp28 clones (Fig. 5B). Taken together, our results are consistent with the conclusion that p28 is not required for efficient infectivity or HTLV-2-mediated immortalization of primary human T-lymphocytes in culture.

### *In vivo *rabbit inoculation results

**Figure 6:**
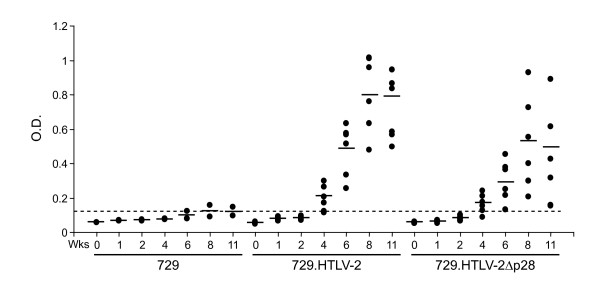
**Assessment of HTLV-2 infection in rabbits**. Antibody response against HTLV-2 from each rabbit was measured by anti-HTLV commercial ELISA assay, using both HTLV Gag and envelope proteins as antigens. Each dot represents the absorbance value of a single inoculated rabbit at 0, 2, 4, 6, 8, and 11 wks post inoculation within each group. Inocula used for the rabbits were 729.HTLV-2 (n = 6), 729.HTLV-2Δp28 (n = 6), or 729 (n = 2). The horizontal line represents the average of the rabbit group at each weekly time point and the dotted line represents three times the standard deviation of uninfected control values.

To evaluate the role of p28 *in vivo*, we compared the abilities of 729, 729.HTLV-2, or 729.HTLV-2Δp28 cell lines to transmit virus to rabbits, which is an established model to investigate HTLV infection and persistence [46]. Rabbits were inoculated with lethally irradiated cell lines (cell inocula were equilibrated based on their p19 Gag production) and on weeks 0, 1, 2, 4, 6, 8, and 11, whole blood was collected and processed for isolation of plasma and PBMCs. Antibody response to viral antigens was detectable by Western blot in all rabbits inoculated with cells expressing either wild type HTLV-2 or HTLV-2Δp28, and the antibody titers in the majority of the rabbits increased over the time course of the study (data not shown). Moreover, quantitative comparison of antibody responses between each rabbit was performed using an HTLV-specific ELISA (Fig. 6). Statistical analysis of titers at six, eight, and eleven weeks post-inoculation revealed a significantly lower antibody response to HTLV-2 antigens in the 729.HTLV-2Δp28-inoculated rabbits as compared to the wild-type HTLV-2 control group. Consistent with our antibody data, HTLV-2 proviral DNA sequences were detected in all wild type HTLV-2 and five of six HTLV-2Δp28-infected rabbits at two weeks post inoculation (Table 1). However, over time, HTLV-2Δp28 failed to persist and quantitative real-time Taqman PCR revealed that at eleven weeks post inoculation, proviral loads in rabbits infected with HTLV-2Δp28 were below the level of detection. Taken together, our results indicated that p28, while dispensable for HTLV-2 infection, attenuated virus replication as measured by antibody response to viral antigens and proviral loads. This attenuation was apparent within two weeks post inoculation, suggesting that p28 is required early for efficient replication and survival in the host.

**Table 1:** Detection of HTLV-2 sequences in PBMCs from inoculated rabbits^a^

	Weeks Post Inoculation				
	
Inoculum and Rabbit	0	2	6	8	11^b^
729.HTLV-2					
R27	-	+	+	-	+ (12.0)
R28	-	+	+	+	+ (8.3)
R29	-	+	+	+	+ (5.3)
R30	-	+	+	+	+ (32.8)
R31	-	+	+	-	+ (10.7)
R32	-	+	+	+/-	+ (4.2)
729.HTLV-2Δp28					
R20	-	+	+/-	+	- (0.2)
R21	-	+/-	-	-	- (0.1)
R22	-	+/-	-	-	- (0.3)
R23	-	+/-	-	-	- (0.3)
R24	-	+/-	-	-	- (1.1)
R25	-	-	-	-	- (1.2)
729					
R1	-	-	-	-	- (0.1)
R6	-	-	-	-	- (0.3)

^a^Genomic DNA was isolated from rabbit PBMCs and subjected to standard PCR (40 cycles) using HTLV-2 specific primers (TRE-pH6-S/TRE-pH6-AS). -, no amplified PCR fragment; +, amplified PCR fragment.

^b^Numbers in parentheses at wk 11 denote copy number per 1000 cells of rabbit PBMC as determined by real-time RT PCR. Copy numbers in rabbits inoculated with 729.HTLV-2Δp28 at wk 11 were significantly different than 729.HTLV-2 as determined by ANOVA followed by Turkey's test (p<0.00032)

## Discussion

The importance of the HTLV-2 nonstructural or accessory proteins in virus biology either in cell culture or in inoculated animals has not been investigated thoroughly. A previous study evaluated an HTLV-2 molecular clone containing a large deletion within the proximal pX region, which at the time was thought to delete the coding sequences for all the known accessory proteins. Results from this study indicated that this region, which later was shown to contain open reading frames (ORFs) for p10 and p11 [28], was dispensable for viral infection and cellular transformation *in vitro *[41]. Subsequently, it was demonstrated that this deletion resulted in reduced proviral load and maintenance of infection *in vivo *[42]. However, the role of the HTLV-2 p28 accessory protein encoded by ORF II located in exon 3 of *tax/rex *was not addressed directly in these studies. We previously demonstrated that exogenously over-expressed p28 functions as a negative regulator of viral replication by binding to and retaining *tax/rex *mRNA in the nucleus, thus repressing Tax and Rex protein production and overall viral gene expression [30,31]. In this study, a site directed mutation was introduced in an infectious clone of HTLV-2 that severely truncated p28 (HTLV-2Δp28) while maintaining the ability of the virus to express other gene products. Subsequently, we examined the expression of p28 and determined its biological significance for the infectivity and immortalization of primary T-lymphocytes in cell culture and viral infectivity and persistence *in vivo*.

Data from our transient transfection studies revealed that, in the context of a proviral clone, the repressive effects of p28 on Tax-mediated transcription and Rex function were not apparent (Fig. 2A &2B). In fact, the loss of p28 resulted in a reproducible, but not significant decrease in Tax activity (75–90%). Consistent with the functional reporter assays, quantitative real-time RT-PCR revealed that the levels of *tax/rex *and *gag/pol *mRNA were not dramatically different in cells transfected with HTLV-2 and HTLV-2Δp28 proviral clones (Fig. 2C). Although we could detect p28 encoding mRNA (approximately 10^3^-10^4 ^total copies per 10^6 ^copies of gapdh), p28 protein was below the limit of detection by Western blot. Due to alternative splicing, p28 has the potential to be expressed from two distinct singly-spliced mRNAs (both of these mRNAs also have the potential to produce the truncated p22/p20rex). Studies by Li and Green showed that these two mRNAs have significantly different expression levels in newly infected PBMCs (10^5 ^vs 10^3 ^copies per 10^6 ^copies of cellular gapdh) [43]. Although nearly impossible to definitively confirm experimentally, we hypothesize that the low copy number mRNA is the primary transcript utilized to encode p28, thus resulting in low protein expression (below our limit of detection). To date, with the exception of the HTLV-1 HBZ protein, none of the HTLV-1 or HTLV-2 accessory proteins have been detected in transfected or infected cells. Interestingly, the mRNA copy number of HBZ in infected cells was 10- to 100-fold higher than the other accessory gene mRNAs, which was consistent with its detection [43]. However, we did confirm that over-expression of p28 from a cDNA expression plasmid, but not Δp28, down-regulated Tax-mediated viral transcription in a dose-dependent manner (Fig 3A). Furthermore, we demonstrated that the repressive effects of p28 on Tax-mediated transcription and Rex activity were not detectable in stable cell lines as represented by variable p19 production less than or equal to wild-type HTLV-2 production levels (Fig 4A). Therefore, we speculate that p28 protein expression is temporally regulated and not expressed following transient proviral DNA plasmid delivery or in stable transfectants and/or a threshold level of p28 is required for the repressive activity.

Results from our short-term proliferation and immortalization assays indicated that the reported repressive effects of the HTLV-2 p28 on Tax and Rex [30,31] were not sufficient to disrupt the capacity of the virus to infect, induce proliferation, and/or immortalize primary T lymphocytes *in vitro *(Fig 5A and 5B). Therefore, similar to the HTLV-1 and other HTLV-2 pX ORF-encoded accessory proteins [38,41,47], p28 appears to be dispensable for efficient viral infectivity, replication and primary T-lymphocyte immortalization capacity *in vitro*.

Based on the efficient infectivity and immortalization of cells *in vitro *and the transient infection observed in 729.HTLV-2Δp28-inoculated rabbits, we hypothesize that the function of p28 and its role in HTLV-2 biology involves early virus/host interactions that may include virus spread and/or survival of the infected cell. We observed reduced proviral load as early as two weeks post inoculation as compared to that in the wild type virus-infected rabbits (Table 1). By four weeks, p28 mutant inoculated rabbits showed a significant reduction in the antibody response to viral gene products, which continued for the duration of the study (Fig. 6). By week eleven, we failed to detect a visible PCR amplified band or realtime PCR proviral loads in all HTLV-2Δp28-inoculated rabbits. All wild-type HTLV-2-inoculated rabbits showed variable but significant proviral loads. To date, p28 has been documented to repress Tax-mediated transcription and Rex activity; based on our results, we speculate that p28 might function in concert with other viral gene products to tightly regulate viral replication and/or influence virus expression in the infected lymphocyte to promote infected cell survival (apoptosis vs cell proliferative signals), viral spread, and establishment of persistent infection. It remains possible that p28 may have multiple activities that function at different stages of the infection process. Future experiments designed to quantitatively assess viral infectivity of rabbits at 1–2 days post inoculation will be required to definitively rule out an early block in infection *in vivo*. Interestingly, the gross phenotype of HTLV-2Δp28 *in vivo *showed significant similarities to HTLV-1 HBZ, p30 and p13 virus mutants. More detailed comparative studies will be required to dissect mechanistic differences which may provide important insight regarding how viral proteins function causing the distinct pathobiology between HTLV-1 and HTLV-2.

## Conclusion

In summary, our data confirmed that over-expression of p28 in cell culture repressed viral gene expression, but in the context of a replicating virus, was completely dispensable for efficient cellular immortalization. Utilizing a rabbit model of infection, these are the first biological studies to demonstrate the critical requirement of the p28 accessory protein in the establishment of HTLV-2 infection *in vivo*. It is likely that p28, as a negative regulator of Tax and Rex, is critical in the temporal regulation of gene expression upon infection and promotes cell survival. This importance is not seen without the selective pressure applied by the presence of a functional immune system. These biological studies have led the way for future studies that are needed to understand the function of p28. Such studies will entail identifying the functional domains of the protein involved in localization, protein interactions, and RNA binding as well as precisely identifying the viral mRNA response element. In addition, gene array studies may provide clues as to whether p28 expression by itself has any direct or indirect cellular effects that facilitate the survival of the T-lymphocyte, the natural target for HTLV infection and cellular transformation.

## Methods

### Cells

293T cells and 729 B cell lines were maintained in Dulbecco's modified Eagle and Iscove medium, respectively, supplemented with 10% fetal bovine serum (FBS), 2 mM glutamine, penicillin (100 U/mL), and streptomycin (100 ug/mL). Human and rabbit peripheral blood mononuclear cells (PBMCs) were isolated using Ficoll Hypaque (Amersham, Piscataway, NJ) and Percoll^® ^(Amersham, Piscataway, NJ), respectively, and cultured in RPMI 1640 medium supplemented with 20% FBS, glutamine and antibiotics as above, plus 10 U/mL of recombinant interleukin-2 (IL-2; Roche Applied Biosciences, Indianapolis, IN).

### Plasmids

The p28 cDNA expression vector (CMV-p28-AU1) and the wild type (wt) HTLV-2 infectious proviral clone (pH6neo) were described previously [30,48]. Using PCR mutagenesis and CMV-p28-AU1 as a template, a single nucleotide mutation (C to A) was introduced in the p28 reading frame. This change (nt 7333 of the pH6neo proviral sequence) resulted in a stop codon in the seventh amino acid (aa) of p28, designated Δp28. This specific mutation was designed to not alter the aa sequence of either Tax or Rex, both of which share overlapping reading frames with p28. The Δp28 mutation expressed in the context of the proviral clone pH6neo, was designated HTLV-2Δp28. The mutation in all mutant plasmids was confirmed by DNA sequencing. The Tax reporter plasmid, LTR-2-Luc, and the transfection efficiency control plasmid, TK-Renilla, were described previously [30,31].

### Transfection, reporter assays, and p19 Gag ELISA

293T cells (2 × 10^5^) were transfected using Lipofectamine^® ^(Invitrogen, Carlsbad, CA) as recommended by the manufacturer. For p28 protein detection, cells were transfected with 1 μg of cDNA expression plasmids and 10 ng of TK-Renilla. Cell lysates were prepared at 48 h post transfection and normalized for transfection efficiency prior to Western blot analysis. To assess the repressive effects of p28 or Δp28 by Tax reporter assays, cells were transfected with 1 μg wtHTLV-2 in the presence or absence of variable concentrations (0.2–0.4 μg) of p28 or Δp28 cDNA expression vector and 0.1 μg of LTR-2-Luc, and 10 ng of TK-Renilla or 1 μg HTLV-2Δp28 and 0.1 μg of LTR-2-Luc, and 10 ng of TK-Renilla. Cell lysates were harvested at 48 h post transfection and dual luciferase activity was measured. The data represent average luciferase activity values after normalization for transfection efficiency for three independent experiments. To generate the 729HTLV-2Δp28 stable transfectant, the proviral plasmid clone containing *neo*^r ^gene was introduced into cells by nucleofection using the Nucleofector kit V (Amaxa Biosystems, Gaithersburg, MD). Stable transfectants containing the desired proviral clone were isolated following incubation in 24-well culture dishes in medium containing 1 mg/ml Geneticin (Gibco, Carlsbad, CA). Following a 4–5 weeks selection period, viable cells were expanded and maintained in culture for further analysis. The well-characterized wtHTLV-2 729 producer cell line (729pH6neo) used in this study was described previously [46,49].

### Western Blot

To detect p28, 50 μg of total cell lysates from transfected cells was separated by SDS-PAGE and transferred to a nitrocellulose membrane (Amersham, Piscataway, NJ). Rabbit polyclonal antibodies against p28 or a monoclonal antibody to AU1 (Covance Research Products, Denver, PA,) was used for p28 detection. Rabbit polyclonal antibody to β-actin (Novus Biological, Littleton, CO) was used as a loading control. Proteins were visualized using the ECL western blotting analysis system (Santa Cruz Biotechnology, Santa Cruz, CA).

### DNA isolation, standard PCR, and Taqman real-time PCR

DNA was isolated from 729 producer cells and rabbit peripheral blood mononuclear cells (PBMCs) using the PURGENE DNA purification system (Gentra, Minneapolis, MN). Rabbit DNA (1 μg) was subjected to a standard 40-cycle PCR amplification for detection of integrated provirus and the product was visualized on a 2% agarose gel stained with ethidium bromide. The primer pair used in the PCR, based on the pH6neo sequence, was TRE-PH-S (5'-^41^GAG TCA TCG ACC CAA AAG G^59^-3') and TRE-PH-AS (5'-^298^TGC GCT TTT ATA GAC TCG GC^279^-3'), which amplified a 257 bp product in the HTLV-2 LTR. Taqman real-time PCR (Applied Biosystems, Foster City, CA) using 500 ng of rabbit DNA and 40 cycle amplification was performed in a 25 ul reaction to quantify the proviral copy number per cell in infected rabbit PBMCs using primers and probes directed towards Gag sequences [43]. The reaction contained 100 ng (25 ng/μL) of each primer and the probe at a concentration of 100 pmol/μL. A standard curve was generated for each run using duplicate samples of log_10 _dilutions of a plasmid containing the Gag sequences. The copy number for each sample was determined from the standard curve, and the copy number per cell for each sample calculated based on the estimate that 1 μg PBMC DNA is equal to 67,300 cells.

### Short-term proliferation and long-term immortalization coculture assays

Short term microtiter proliferation assays were performed as detailed previously with modifications [45,50]. Briefly, freshly isolated human PBMCs were pre-stimulated with 2 μg/ml PHA and 10 U/ml IL-2 (Roche, Indianapolis, IN) for three days. 729 producer cells (2 × 10^3^) were irradiated (100 Gy) and co-cultured with 10^4 ^pre-stimulated PBMCs in the presence of IL-2 in 96-well round bottom plates. Wells were enumerated for growth and split 1:3 at weekly intervals. Cell proliferation was confirmed by MTS assay using CellTiter 96^® ^Aqueous One Solution Reagent as recommended by the manufacturer (Promega, Madison, WI). For the long-term immortalization assays, 10^6 ^irradiated producer cells were co-cultivated with 2 × 10^6 ^freshly isolated PBMCs with 10 U/ml IL-2 in 24-well culture plates [41]. HTLV expression was confirmed by detection of p19 Gag protein in the culture supernatant measured at weekly intervals using a commercially available ELISA (Zeptometrix, Buffalo, NY). Viable cells were counted weekly by trypan blue exclusion. Cells inoculated with HTLV-2 that continued to produce p19 Gag antigen and proliferate 12 weeks post co-culture in the presence of exogenous interleukin-2 (IL-2) were identified as HTLV immortalized. For each assay, at least three independent experiments were performed using PBMCs from distinct healthy donors.

### Rabbit inoculation, ex vivo culture, and serologic analysis

Twelve week-old specific pathogen-free New Zealand White rabbits (Harlan, Indianapolis, IN) were inoculated with approximately 1 × 10^7 ^gamma-irradiated (100 Gy) 729 viral producer cells (6 rabbits per group) or 729 uninfected control cells (2 rabbits) via the lateral ear vein. The virus-containing inocula were equilibrated based on HTLV-2 p19 Gag production (ELISA). At weeks 0, 1, 2, 4, 6, 8, and 11 after inoculation, 10 ml of blood was drawn from the central auricular artery from each animal and rabbit plasma and PBMCs were isolated. HTLV Western blot assay (HTLV Blot 2.4 Western Blot Assay; MP Diagnostics, Singapore) was used to examine serum reactivity to specific viral antigenic determinants. Serum showing reactivity to Gag (p24 or p19) and Env (gp21 or gp46) antigens was classified as positive for HTLV-2 seroreactivity. A commercial HTLV ELISA kit (Vironostika HTLV-I/II Microelisa System; bioMerieux, Durham, NC) was used to quantitate HTLV-2 serum antibody using plasma diluted 1:100 to obtain values within the linear range of the assay. Data is shown as absorbance values. DNA was isolated from rabbit PBMCs using the PURGENE DNA purification system (Gentra, Minneapolis, MN) and subjected to proviral load analysis by realtime PCR.

## Authors' contributions

BY generated mutant clones, carried out functional assays, virus replication and immortalization assays, the *in vivo *studies, and drafted the manuscript. ML developed the realtime PCR primers and performed or assisted with all the assays and quantitation. MK helped with the collection and processing of *in vivo *samples, and assisted with the Western blot analysis. IY helped with the generation of mutant clones and the development of the functional assays. MDL has helped in finalizing the manuscript and has provided important input on the design of the rabbit portion of the study. PLG conceived the study, participated in its coordination, helped in drafting and finalizing the manuscript. All authors read and approved the final manuscript.
